# Categorial modal realism

**DOI:** 10.1007/s11229-023-04042-4

**Published:** 2023-01-30

**Authors:** Tyler D. P. Brunet

**Affiliations:** 1 Egenis, The Center for the Study of the Life Sciences, University of Exeter, Byrne House, St German’s Road, Exeter, Devon EX4 4PJ UK; 2 Department Social and Political Sciences, Philosophy and Anthropology, University of Exeter, Amory Building, Rennes Drive, Exeter, EX4 4RJ UK

**Keywords:** Modality, Possible worlds, Category theory, Modal logic, Humility

## Abstract

The current conception of the plurality of worlds is founded on a set theoretic understanding of possibilia. This paper provides an alternative category theoretic conception and argues that it is at least as serviceable for our understanding of possibilia. In addition to or instead of the notion of possibilia conceived as possible objects or possible individuals, this alternative to set theoretic modal realism requires the notion of possible morphisms, conceived as possible changes, processes or transformations. To support this alternative conception of the plurality of worlds, I provide two examples where a category theoretic account can do work traditionally done by the set theoretic account: one on modal logic and another on paradoxes of size. I argue that the categorial account works at least as well as the set theoretic account, and moreover suggest that it has something to add in each case: it makes apparent avenues of inquiry that were obscured, if not invisible, on the set theoretic account. I conclude with a plea for epistemological humility about our acceptance of either a category-like or set-like realist ontology of modality.

## Introduction

Is there a *set* of possible worlds? This question is importantly different from whether there *are* possible worlds, since it can be appropriately rephrased as follows: Is the plurality of worlds *a set* of possible worlds? This essay argues that we do not need to be committed to a set of possible worlds to be modal realists, that we do not need to think the plurality of worlds is a set or set-like, since there is a viable alternative: that there is a collection of possible worlds and possible morphisms. That is, we can be modal realists by believing that there is a *category* of possible worlds, that the plurality of worlds is a category, or at least sufficiently category-like. This essay describes and argues for this categorial alternative to modal realism (Sects. 2–3), then shows how it meets two desiderata of an account of modality: that it can provide a basis for modal logic (Sect. 4.1), and that it can handle a size-based objection to the plurality of worlds (Sect. 4.2). I conclude with the idea that a category theoretic modal realism is a metaphysics of modality that can contend with Lewis’ set theoretic modal realism on Quinean grounds.

Lewis is explicitly committed to the claim that there is a set of worlds. Lewis often refers to “the set of possible worlds.” That alone might be charitably read as indirect, non-technical, non-committal, speech—something that does not commit Lewis to the view that there really is a set of possible worlds nor to the view that the plurality of worlds is a set of worlds. However, Lewis also states directly that he accepts a set of all worlds, in his arguments against Forrest and Armstrong’s (1984) size-based objection to his modal realism (more in Sect. 4.2). Lewis resolves this objection by placing a proviso on how large individual worlds can be. However, he notices a loophole in Forrest and Armstrong’s *reductio* of modal realism that turns on whether the plaurality of worlds is a set, saying “[I]f there are the worlds, but there is no set or aggregate of all of them, then the contradiction is dodged. Does this loophole give me a way to do without the unwelcome proviso? I think not” (Lewis 1986, p. 104).

Among the reasons he gives against taking this loophole are that some uses of *possibilia* “will require the forbidden sets” [ibid]. However, even if one can provide for these uses without sets, Lewis has a more serious commitment to a set of all worlds,

How could the worlds possibly fail to comprise a set? [T]he obstacle to sethood is that the members of the class are not yet all present at any rank of the iterative hierarchy. But all the individuals, no matter how many there be, get in already on the ground floor. So, after all, we have no notion what could stop any class of individuals—in particular, the class of all worlds—from comprising a set. Likewise we have no notion what could stop a class of individuals from comprising an aggregate. So I continue to accept a set of all worlds, indeed a set of all individuals.—Lewis (1986, p. 104)

In this case it is clear that Lewis is using ‘set’ in its technical sense, that he does not hold an alternative to the plurality as a set, and that he advances modal realism as a theory while accepting a set of all worlds. Indeed, we do have a notion that can stop the plurality of worlds from comprising a set: the notion of a category. Categories may be founded on sets and they may contain sets, but they are not, in general, sets.^1^ Sets may be thought of as unstructured collections, while categories should be conceived as having additional structure, embodied in their network of morphisms (see Sect. 3).

In Sect. 2 I argue that the plausible equality of category theory and set theory in meeting the fundamental needs of mathematics changes Lewis’ analogy between the plurality of worlds and the universe of sets as paradises for intellectual activity. In Sect. 3 I sketch a verbal formulation of category-like modal realism, stressing the importance and utility of the role of *possible morphisms* in addition to or instead of possible worlds and possible individuals. Finally Sect. 4 offers two example cases where a categorial modal realism can be put to work where a set-like modal realism has so far been prominent. These are (Sect. 4.1) how category-like models can be used in place of Kripke-models and counterpart-models in modal logic, and (Sect. 4.2) how a categorial approach can handle size-based objections to the plurality of worlds. To be clear, this essay does not argue that the categorial approach is necessarily an improvement over the set theoretic—I suspect it is, in some respects, though do not argue for that here. Instead I argue that the two are at least on par with respect to some core theoretical desiderata, and so the categorial approach should be considered a contender to be our metaphysical account of modality.

## Two paradises of equal benefit

I begin with a somewhat unfair tactic. I argue that an offhand remark justifying an analogy made by Lewis—within a footnote—is not entirely correct, and that this has profound consequences for his overall view. That remark is the following, the analogy appears below.

Why believe in a plurality of worlds?—Because the hypothesis is serviceable, and that is a reason to think that it is true... Hilbert called the set-theoretical universe a paradise for mathematicians... We have only to believe in the vast hierarchy of sets, and there we find entities suited to meet the needs of all the branches of mathematics [footnote: With the alleged exception of category theory—but here I wonder if the unmet needs have more to do with the motivational talk than with the real mathematics.].—Lewis (1986)

This remark serves as the basis of Lewis’ justification for belief in a plurality of worlds by analogy to the utility of belief in a vast hierarchy of sets—both being Quinean desert landscapes in Lewis’ view. However, the dismissal of the exceptionalness of category theory is substantial and hasty. A better view of the relationship between set theory and category theory suggests a different analogy and a different paradise for philosophy.

Category theory is not an exception, but the category theoretic universe is *also* a paradise for mathematicians. In parallel with the belief that the plurality of worlds is like a universe of sets, this more amenable view of category theory in mathematics suggests that it is also serviceable to believe that the plurality of worlds is like a category.

We can unpack Lewis’ remark about category theory and set theory as follows: it is allegedly the case that meeting the needs of category theory as a branch of mathematics will require something more or other than the vast hierarchy of sets, but this only appears so due to the way that category and set theorists talk; that motivational talk properly reformed, the hierarchy of sets meets the needs of category theory. This is a common view, although there is a growing consensus that it is not entirely correct. Here is a two-step rejoinder: the needs of category theory *can* be met by set theory, but the needs of set theory can also be met by category theory (Lawvere, 1966; Mac Lane, 1969; Landry & Marquis, 2005; Landry, 2011; c.f Mayberry, 1994). The two are contenders for meeting the needs of all branches of mathematics.

Lewis admits that the utility of set theory is a “good” but not “conclusive” reason to believe its ontological commitments. For a reason that it is not conclusive he cites, *inter alia*, the option that “perhaps some better paradise might be found” (p. 4). I do not find any conclusive reasons that category theory is better, however there are plenty good reasons to think that it is at least as good. Category theory has been successful in meeting the foundational needs of mathematics and in engendering new needs and connecting distant branches. As a “tool in the mathematician’s toolbox” (Marquis, 2021) its utility has been that it “organizes and unifies” [ibid] distant problems, including those at foundational levels. This is reason enough to think that it is, like set theory, a theory fit for foundations. The hypothesis that there are vast categories is perhaps not more serviceable, but it is serviceable, and this is a good reason to think that it is true.

It is also not conclusive. As Lewis (1986, p. 4) points out for set theory, perhaps category theory has “unacceptable hidden implications”, so that a round of category-theoretical paradoxes will soon be upon us. Perhaps accepting controversial ontology for theoretical benefits is wrong, as a sceptical epistemologist might say. Perhaps paradise better still might be found, or some mathematical activity discovered, the needs of which can only be provided for set-theoretically. Perhaps we might even find a way to accept category theory without an ontological commitments to categories (or, to objects and morphisms). The point remains: category theorists have also found it worth believing in “vast realms of controversial entities for the sake of enough benefit in utility and economy of theory” (Lewis, 1986 p. 4). Some philosophers might like to see it otherwise, but working mathematicians insist on pursuing their subject.

This plausible parity of set theory and category theory affects the following analogy, where the second sentence is justified in part by the first.

As the realm of sets is for mathematicians, so logical space is a paradise for philosophers. We have only to believe in the vast realm of *possibilia*, and there we find what we need to advance our endeavours.—Lewis (1986, p. 4)

I suggest we take the analogy at face value while denying that the realm of sets is for mathematicians the bargain that Lewis assumed. The realm of sets is not the cheapest ontology at the greatest benefit, but one of two equally priced ontologies with the same benefits. Read this way, the analogy says that logical space is one of two coequal paradises for philosophers. The analogy still justifies the claim that belief in a ‘vast realm of *possibilia*’ is sufficient for the needs of philosophical endeavours, but no longer justifies the claim that this vast realm is necessarily set-like.

Goldblatt said that today’s pathology may one day be dubbed “classical” by future mathematicians ((, 1984), p. xii). Today, worries about the pathology of category theory as an exception have succumbed to by-now classical categorial foundations. Today, Hilbert might have said that mathematics does not have a unique paradise, but two coequal paradises. So too for philosophy. Categories are also a good source of analogies for our ontological commitments in philosophy; a category-like logical space is also a paradise for philosophers.

Lewis does not exclude the possibility of alternatives to his view. In philosophy as in mathematics, justification for belief in vast realms on the basis of their utility is not “conclusive” reason.

Maybe—and this is the doubt that most interests me—the benefits are not worth the cost, because they can be had more cheaply elsewhere.—Lewis (1986, p. 5)

The alternatives to his modal realism that he considers, for purchase of the philosophical benefits elsewhere, are what he and others have seen as modal *ersatzisms* (“linguistic”, “pictoral” and “magical” varieties of non-realist or anti-realist theories of modality). He finds these alternatives wanting, and for good reason so far as I can tell. What he does not consider are alternatives to his view that are equally “realist” and equally “vast”.

The remainder of this essay develops this unconsidered alternative. There are different sorts of “vast realms” in logical space. For present discussion I assume the only relevant differences are between those which are *set-like* and those that are *category-like*, and between those that are *realist* and those that are *ersatz*. Lewis argued for a realist set-like vast realm by arguing for its utility and by arguing against an ersatz set-like realm. I argue for a realist category-like vast realm by showing it coequal to a realist set-like realm. The next Sect. (3) describes the approach and the following Sects. (4.1–4.2) shows how it handles two desiderata of a theory of modality.

## The essentials of categorial modal realism: possible morphisms

To develop a categorial alternative to set-like modal realism, this section will argue that when considering the plurality of worlds or “logical space” we should consider not only the *possibilia*, the possible individuals, but also their possible transformations, processes or changes. The most mathematically well-developed way to do this is using category theory. Categories are presented^2^ as collections of two sorts of things: a collection of objects and a collection of morphisms.^3^ By analogy, categorial modal realism is a belief in a plurality of possible objects and a plurality of *possible morphisms*. Leaving possible objects (individuals or worlds) mostly as they are, this section explores the utility of appending an account of possible morphisms.

The notion of a *morphism* is a generalization of the idea of a *homomorphism* from abstract algebra. Homomorphisms between algebras preserve algebraic structure. For example, a group-homomorphism between two groups preserves group structure.^4^ Morphisms (also called arrows, functions, or maps) between objects of a category are usually defined by their preservation of some key property (usually specified by the name of the category or morphism). The category of topological spaces has continuous set-functions as morphisms, i.e. functions that preserve openness of sets; the category of pointed sets has functions that preserve pointedness as morphisms^5^; the category of partially ordered sets has monotonic (order preserving) maps as morphisms. In many categories the morphisms will be described as set-theoretic functions of some sort, however morphisms need not be functions between sets. The category of relations has sets *X*, *Y*, *Z*, ... as objects but *relations* as morphisms (i.e. $R \subseteq X \times Y$), and only some of the relations are functions; abstract categories can also be specified just by the network of their morphisms, without explicitly specifying either a preserved property or function.

Formal specification of a morphism must include its domain (what it is a change from) and its codomain (what it is a change to) as well as its sort (what is preserved). The domain of a morphism $f: x \rightarrow y$ is denoted *dom*(*f*) (here, $dom(f) =x$) while the codomain is denoted *cod*(*f*). If we think of the domain and codomain of a morphism as having a type (e.g., sets, groups, rings), then then morphism preserves their type. A morphism $f:x \rightarrow y$, is $\phi$-preserving iff $\phi (x) \implies \phi (f(x))$, where $\phi$ is some interesting property of objects of the type of *x*, and typically involves quantification over the elements of *x*. For a category $\mathfrak {C}$ the objects of that category are denoted $obj(\mathfrak {C})$ and the morphisms or “arrows” by $arr(\mathfrak {C})$. To comprise a category, a collection of objects and morphisms of $\mathfrak {C}$ must additionally satisfy the *category axioms*.

1. **Existence of Composites**: For every pair of morphisms $f: x \rightarrow y$ and $g: y \rightarrow z$, such that $cod(f) = dom(g)$, there exists a morphisms $g \circ f: x \rightarrow z$ called the composite of *g* with *f*.
2. **Associativity of Composition**: $f \circ (g \circ h) = (f \circ g) \circ h$ whenever such composites are defined.
3. **Existence of Identities**: For every object $x \in obj(\mathfrak {C})$ there is a morphism $Id_{x} \in arr(\mathfrak {C})$, such that, $Id_{x} \circ f = f: y \rightarrow x$ and $g \circ Id_{x} = g: x \rightarrow y$, called the identity morphism for *x*.

The attitude of categorists, when considering a newly defined object, is to immediately ask: In a category of these objects, what are the morphisms? Since we have defined categories, we should define their morphisms. In the category of categories **Cat**, the morphisms $F: \mathfrak {C} \rightarrow \mathfrak {D}$ between categories are *functors*. Remarkably, attempts have been made to treat functors as a primitive notion in a direct axiomatization of **Cat** (McLarty, 1991; Blanc & Preller, 1975; Lawvere, 1966). For our purposes, it is more convenient to define functors $F: \mathfrak {C} \rightarrow \mathfrak {D}$ on the basis of a pair of morphisms,^6^ (both noted the same) $F: obj(\mathfrak {C}) \rightarrow obj(\mathfrak {D})$ and $F: arr(\mathfrak {C}) \rightarrow arr(\mathfrak {D})$, satisfying two conditions.

1. **Preservation of Identities**: $F(Id_{c}) = Id_{F(c)}$ for every object $c \in obj(\mathfrak {C})$.
2. **Preservation of Composition**: $F(f \circ g) = F(f) \circ F(g)$ for every composable pair $f,g \in arr(\mathfrak {C})$.

This is all of category theory we will use in this section.^7^

This section argues that it is also fruitful to apply notions analogous to morphism and category to non-mathematical objects. In the philosophical context, objects (in the broadest sense) sit within ontological categories (such as person, substance, place or world). The morphisms of these objects are just their changes (in the broadest sense) where some property is preserved. A change of a person is a personhood morphism iff the change is personhood-preserving (the change does not affect their personhood). Moreover, a change is a personal identity morphism iff it is personal identity preserving (it is a change between two instances of the same person). A change (e.g. of shape) acting on an object is a substance morphism iff it preserves the substance of the object (e.g. does not affect atomic number). A change is a world morphism iff it preserves worldhood (the absence of extra-worldly st-relations). Perhaps every change occurs within a world and nothing can participate in extra-worldly st-relations. If so, every change is a world morphism. Moreover it is fruitful to assume that the collection of such morphisms of an ontological category (in the philosophical sense) is—for the category *worlds* especially (see Sect. 4.1)—a rich enough structure to satisfy the axioms for being a category (in the mathematical sense).^8^

Here are some examples of morphisms in greater detail. A change from one person, say ‘David at age 10’, to (potentially) another person ‘David at age 20’ is a personal identity-preserving morphism iff ‘David’ is the same person at both ages. We might likewise specify a continuous personal identity morphism as one that preserves personal identity at each and every moment in time during the decade, or of each and every temporal-part of David. The (actual) change from ourselves at an earlier age to ourselves now is a personal identity preserving morphism—though it may preserve little else. Biological death is not a personal identity preserving change, so it is not a morphism of persons. Ovidian metamorphoses are morphisms of various sorts. Athena’s transformation of Medusa into a monster is a psychological-identity preserving change, while Apollo’s transformation of Daphne into a tree apparently only preserves terror. The change from a caterpillar to a butterfly must preserve organismal identity to be a metamorphosis in the entomological sense.

Perhaps identity must be preserved for there to be change of something at all, or perhaps there must be numerical identity for there to be qualitative changes, perhaps there must be essential natures for there to be accidental changes, perhaps there must be haecceities, substances or monads for descriptions of change to refer. If so, then every change is a morphism of some sort. If not, then the morphisms are a restricted class of the changes. Either way, we have a usable concept that covers a variety of familiar changes—and probably some unfamiliar ones as well. Many actual morphisms of ontological kinds are familiar cases in which a change preserves some ontologically relevant property; I ask the reader to assume that there are many non-actual morphisms as well.

What does any of this have to do with modality? A great deal of our alethic claims are about possible changes. When we consider whether Hillary could have won the election, a good way to interpret this is as about whether Hillary prior to the election could have changed into Hillary after the election, keeping her personal identity while changing title. When we ask whether a caterpillar could fly, we are probably not asking if it has hidden wings or whether caterpillars fly without them. We are asking whether it could metamorphose.

In informal English reasoning about modality, we often express the possibility of one state of affairs by reference to another state and the existence of a possible change from the other to the one. Perhaps all possibility claims can be analysed like this. We can elaborate on the claim that Hillary could have won the election by saying that there was, at one point, a *way* or *path* to victory. I *could* have a sandwich for lunch if *there is a way* for me to get a sandwich by lunchtime; I *couldn’t* have soup for lunch if *there is no way* for me to get soup by lunchtime. Traditional alchemy is impossible since there *is no way* to transmute lead into gold. I take possible-ways and possible-paths to be flavours of possible-change and—when these involve preservation of properties such as my personal identity during a sandwich-hunt or the nuclear integrity of atoms during a chemical reation—they provide instances of reducing alethic modal claims to those asserting the existence of possible-morphisms. We will see that this can be made precise in Sect. 4.1.

At this point we should forestall an objection to the ontological status of possible morphisms. The objection runs like this: possible morphisms are mere (individual) changes in some possible world, so are already covered by set-like modal realism. I can see no reason to deny that some of the possible morphisms correspond 1–1 with a class of *possibilia* in worlds, although I do not see this as a concession to a set-like vast realm. Indeed, the contrary can also be adopted: that all possible individuals are possible morphisms, so that category-like modal realism also covers the class of *possibilia*. One of the first lessons from Eilenberg and Mac Lane’s (1945) original treatment of category-theory is that granted weak axioms about (1) the existence of identity mappings for each object of a category and (2) objects for each identity mapping, we can theoretically do away with objects. They say,

These two axioms [provide] a one-to-one correspondence between the set of all objects of the category and the set of all its identities. It is thus clear that the objects play a secondary role, and could be entirely omitted from the definition of a category. However, the manipulation of the applications would be slightly less convenient were this done.—Eilenberg and Mac Lane (1945, p. 238)

Analogously, by assuming that there is an identity morphism for each individual—one that preserves everything about that individual—and that there is an individual for every such morphism, we can just as well adopt the contrary view that possible individuals play the secondary role. *Possibilia* could be entirely omitted. Moreover, granted that we can identify each possible world with a sort of trivial identity morphism of worlds, and every such identity with a world, then we can extend this conclusion about possible individuals up to the level of worlds and claim that they also play a secondary role and can be omitted from our definitions of modality. In Sect. 4.1 we will see that this carries over to modal logic: when categories are used as models, we can eliminate reference to possible worlds in the definitions of the truth conditions for the usual modalities. Why do I not take this line here, since it would indeed more clearly display the autonomy of the categorial approach? Because it is convenient to separate the roles of object and morphisms, and that is a good reason to separate them.

Moreover, there are still the non-identity morphisms left over after we draw up a correspondence between identity morphisms and individuals. What if the set-like realist claims that these too can be paired up with individuals in some possible world? Again I can see no reason to deny it, though it is little concession to a set-like vast realm that a possible morphism is, in some world, a possible individual. Indeed it makes higher-order claims about morphisms more convenient to state clearly. If the possible morphism *f* at *w* can be associated with a possible individual $f'$ at $w'$, then the morphisms of $f'$ at $w'$ are higher-order possible morphisms for the individuals at *w*.^9^ It is no trouble for the vast realm of *possibilia* to include possible individuals for non-identity morphisms, so long as this is done in a way that is serviceable (e.g. to higher-order modal claims).

For example, a caterpillar could fly iff there is an organism-identity preserving possible morphism between a counterpart individual caterpillar and a flying thing (e.g. a butterfly). What if this possible morphism of individuals is itself an individual metamorphosis in some world?^10^ Then it could be domain or codomain for higher-order morphisms. For an example of a higher-order morphism, a metamorphosis could have occurred without a high-sugar diet iff there is a life-cycle preserving morphism from a high-sugar metamorphosis^11^ to a low-sugar metamorphosis.^12^ From the standpoint of our world, this amounts to a higher-order morphism between morphisms even though it is more conveniently described as a possible morphism between individual metamorphoses. For instance, as a morphism that preserves the development of metamorphosis while changing the course of evolutionary events to one where caterpillars eat only lipids. That, indeed, is not an individual in our world—our world does not, so far as we know, contain these sorts of lateral historical changes—but it might harmlessly be treated as an individual in another world.

Notice that the notion of *preservation* in morphisms parallels the idea of *accessibility* by a relation. Importantly, $\phi$-preservation can determine the sort of modality under consideration. The most well-to-do use of possible worlds is in transforming modality into *restricted* quantification, where restriction is achieved by accessibility relations. For instance, defining the nomological modalities, *A* is nomologically necessary iff *A* is true at *every nomologically accessible* world. A world is nomologically accessible from our world iff it “obeys the laws” of our world. Similarly, a world is “historically accessible” iff it “perfectly matches ours up to now” (Lewis, 1986, p. 7). This is a *façon de parler* that we have inherited, but it is not the only one. We also sometimes talk of “shifting” our attention to another world where some condition holds, or of “jumping” to the closest such world (see the letter from Geach to Prior, April 15, 1960, cited in Copeland 2002). “Shifting” or “jumping” between worlds is a sort of change, and Copeland (2002) makes the case that our use of ‘accessibility’ historically derives from the literal sense (a possible change of location) imagined by Geach as a process of jumping between worlds. I add that we can say the same things—perhaps even say them more naturally—in terms of morphisms instead of relations.

Taking the morphism route here perhaps even affords us a small bit of economy in theory. We can still define modalities by restricted quantification, but can define restriction directly in terms of preservation, instead of defining a relation between worlds, itself defined by preservation. Of course, noticing that accessibility relations tend to be defined by the preservation of some $\phi$, we could have always done things this way, but the language of sets and relations obscures this option somewhat. For example, defining nomological modalities, *A* is nomologically necessary iff *A* is accessible from every world-law-preserving morphism. Likewise *A* is historically necessary iff *A* is accessible from every world-history-preserving morphism. For a first-order counterpart example, it is anthropologically necessary that David is human iff all of the individuals accessible by David’s-identity preserving morphisms are human—or, for a morphisms-only definition with even greater economy—iff all of the David’s identity-preserving morphisms are also humanity-preserving.

With the resources introduced so far, we are able to discuss individuals of various ontological categories and their morphisms and translate many alethic claims about them into claims about the existence of possible morphisms. To do this above I treated it as unproblematic to discuss possible morphisms of individuals at our world (e.g. Hillary, a caterpillar, etc.). However, in a set-like modal realist context such translations do encounter philosophical problems, since they often require reference to contentiously related otherworldly individuals (e.g. Hillary herself, except in another world, or a counterpart of Hillary). That is, these sorts of alethic claims about individuals at a world encounter problems of deciding on an account of transworld identity or counterpart relations (see review in Mackie & Jago, 2018). In the remainder of this section I argue that a categorial approach to mapping individuals between ontological categories, based on functors, is sufficient to provide for both identity and counterpart based approaches to alethic claims about individuals.

For present purposes, an identity theory of otherworldly individuals is any that treats it as unproblematic (or somehow resolved) to treat some otherworldly individuals as literally *identical* to some this-worldly individuals. Counterpart theories are any that instead deploy a *relation* between this-worldly and otherworldly individuals. Counterpart theory is due to Lewis (1968), who attributes identity theories to Carnap and Kripke (*inter alia*). In his words, “The counterpart relation is our substitute for identity between things in different worlds ” (Lewis, 1968, p. 114). In my view the best summary and most important theoretical elaboration of counterpart theory was provided in Lewis (1971). Here is the summary.

To say that something here in our actual world is such that it might have done so-and-so is not to say that there is a possible world in which that thing itself does so-and-so, but that there is a world in which a counterpart of that thing does so-and-so... the counterpart relation is one of similarity.—Lewis (1971)

The elaboration—intended to deal with problems of personal and bodily identity—was to allow for a “multiplicity of counterpart relations”.

In certain modal predications, the appropriate counterpart relation is selected not by the subject term but by a special clause. To say that something, regarded as a such-and-such [e.g. as a body or as a person], is such that it might have done so-and-so is to say that in some world it has a such-and-such-counterpart that does so-and-so.—Lewis (1971, p. 210)

I will now argue, in service of a categorial modal realist position, that functors between ontological categories suffice for both identity theory and Lewis’ elaboration of counterpart theory.

Recently Varzi (2020, p. 4693) argued that identity and counterpart theory are “two species of the same genus, two distinguished special cases of an otherwise uniform semantic framework” by showing that both can by obtained by translating modal claims into a sufficiently general language in standard extensional predicate logic with a variable counterpart relation—one allowed, under assumptions congenial to identity theorists, to be the identity relation. My approach is similar, though formulated with general functors instead of (counterpart) relations. I prefer this approach because it coheres best with the assumption that individuals exist in ontological categories with sufficient structure to satisfy the conditions for being a mathematical category, and because it adds a bit of generality without losing any of the expressive capacity available from relations.

Lewis was insistent that the counterpart relation be one of similarity. This is a requirement for his theory because, in his set-like plurality, similarity is the only plausible connection between the properties of individuals in distinct worlds. However, within a category-like plurality equipped with possible world-morphisms, another connection becomes available: one individual can be the image of another according to some specified sort of world-morphism. Since we are thinking of the contents of worlds as ontological categories, the morphisms required to preserve these categorial structures are functors. Again this can give us a small bit of economy in theory, since (under some conditions) the world-morphisms that serve as our substitute for relations of accessibility may *also* serve as our substitute for relations of counterparthood.

Here is the general description, in line with Lewis’s summary above: To say that something here in our actual world is such that it might have done so-and-so is not to say that there is a possible world in which *that thing itself* does so-and-so, but to say that there is a world-morphism (the codomain of which is thereby an accessible world) on which *the image of that thing* does so-and-so. Moreover, this framework also allows a direct and succinct substitute for Lewis’ elaboration to a multiplicity of counterpart relations, as follows: In certain modal predications, the appropriate counterpart is selected by a special clause. To say that something, regarded as a such-and-such (e.g. a human, person, organism), is such that it might have done so-and-so is to say that there is a world-morphism that, when restricted to that something, is a such-and-suchness preserving morphism and the image of that something does so-and-so. On this account, relevant counterparts indeed must be similar in certain respects, but they are not counterparts because they are similar, they are similar because the counterpart morphisms that determine them must preserve some of their relevant properties (e.g humanity, personhood, organismal identity etc.). For example, I have a human-counterpart iff there is a possible way to transform the actual world into another, so that the transformation acting on myself preserves my humanness.

Since functors are defined on the entirety of their domain category and must, like functions, give unique outputs for each input, it might seem as if we have excessively restricted counterparts by using functors, by comparison with giving counterparts by relations (which have no such constraints). This is not the case and we can see so with a few examples covering some standard unusual counterpart scenarios. What if I have *no counterparts* at a world? That functors (world-morphisms) must give *some* image for each object in their domain might seem to imply that, if a world is accessible at all by that functor, then I must have some counterpart there. However, the special clause takes care of this. It may be the case that I have no $\phi$-counterpart at some accessible world if there is no world-morphism between them which, when restricted to its action on myself, is $\phi$-preserving (preserving of whatever way in which I am thinking of myself as having a special sort of counterpart). My image on some world-morphism may be an amorphous lump, and such a lump is not one of my personal-counterparts.

What about twinning? That functors have unique outputs might seem to imply that I cannot have two, or more, counterparts at another world. However, nothing about the use of functors implies that the image of an individual on some world-morphism cannot have any additional structure, e.g., the structure of a set or mereological sum. Here is an imaginable world-morphism: The world’s tape rewinds to a time when I was a zygote, then continues again, progressing along an historical path where that zygote splits into a pair of identical twins. Let us assume that the image of myself along this morphism is one of these twins in particular. That is no problem for functors, and this gives a clear sense in which *I could have had a twin*, since I have a counterpart with a twin. But it is also no matter to suppose that my image on this world-morphism is the pair (or sum) of twins. On the assumption that my image on this world-morphism is the pair of twins, there is a clear sense in which *I could have been twins*. There is nothing problematic about my having an individual or collection of counterparts, though, as with the case of my having a twin vs. my being twins, I think the individual counterpart case is typically what is meant.

It should help to examine why, on morphisms, it makes sense for their counterparts to be given functorially. Firstly, if $f:c \rightarrow c'$ is a morphism between individuals of some ontological category, then a counterpart of this morphism—let me call it a ‘countermorphism’—must be a morphism $F(f): F(c) \rightarrow F(c')$ between the counterparts of those individuals. This is a constraint imposed by giving counterparts functorially, but it is a constraint we should adopt. We would not want, e.g., the countermorphism of the process of my (actual) failing to get a sandwich by lunchtime to be a morphism of some non-counterpart of me (say a counterpart of my coworker) succeeding to get a sandwich by lunchtime—that would not assure me that *I* could have got one. Similarly for the constraint that counterpart functors should preserve composition. If $f \circ g:c \rightarrow c' \rightarrow c''$ is a composite of two morphisms $g: c \rightarrow c'$ and $f: c' \rightarrow c''$ between individuals, giving its countermorphism functorially means that it must be a composite of the countermorphisms of the components, i.e., $F(f \circ g) = F(f) \circ F(g)$. This constraint again makes sense when we are thinking of individuals at worlds as comprising ontological categories. Consider a composite, e.g., (*f*) Hillary failing to institute progressive vaccine policies ($\circ$) after (*g*) Hillary losing the election. To say that it was possible for Hillary to institute progressive vaccine policies after winning the election is to claim that there is a countermorphism of this composite which is an institution of progressive vaccine policies after winning the election. By the first condition, it must be a countermorphism of a counterpart of Hillary, and by the second it must be a composite of the countermorphisms of *f* and *g*. If it were not—suppose for example it was some other composite ($F(f) \circ F(h)$) of the countermorphism of a counterpart of Hillary instituting progressive vaccine policies (*f*) composed with a countermorphism of a counterpart of someone else losing the election ($h \ne g$)—then I cannot see how this would assure me that Hillary could have undergone that composite of changes.

This section has introduced the notion of morphisms between ontological categories and argued for their utility in providing an account of alethic modal claims. The next (Sects. 4.1–4.2) will put these notions to work. I ask the reader to assume—I think, not onerously—that ontological categories and their category-preserving changes are sufficiently rich to satisfy the category axioms. At least, it is useful to assume this about some common ontological categories, such as person, place, thing/process, and world. With notions of morphisms of individuals and worlds in hand, we can do much. World morphisms can serve instead of accessibility relations in defining types of modality and can be used to give counterparts of individuals at accessible worlds. This is explored further in Sect. 4.1. In Sect. 4.2 I will argue for another use: *isomorphisms* of worlds can help resolve pernicious paradoxes related to the size of the plurality.

## Categorial modal realism at work

The sceptic realist might wonder why to bother with morphisms when *possibilia* as individuals—and worlds thereof—seem to meet our needs with abundance. My answer is that both are fruitful ontologies, but that it is also fruitful sometimes to shift our ontological perspective. To satisfy the modal realist who believes in a set-like vast realm of *possibilia*, I discuss two ways that a category-like realm can satisfy some of the desiderata of a metaphysics of modality, while perhaps making some interesting avenues of inquiry more apparent.

In Sect. 4.1 below I show how (pointed) categories can be used just as well in place of Kripke models in a semantics of familiar modal logics (**S4**, **S5**). Indeed, the two sorts of models are not equivalent. This is the interesting point about the shift in perspective: they are “weakly equivalent”, since the category of pointed categories is adjoint to the category of Kripke models. I then show how a quantified modal logic can just as well be based on models using counterpart functors. In Sect. 4.2 I show how a categorial approach can block the Forrest-Armstrong paradox similarly to Lewis’ own resolution. This approach is based on world-isomorphisms to an ontological analogue of Grothendieck universes, lending itself naturally to a conception of large worlds and large pluralities of worlds.

### Modal logic: arrows instead of accessibility relations

2 of ways to categorify the standard Kripke semantics for modal logic (Goldblatt, 1981; Kishida, 2011, 2017; Awodey & Kishida, 2008; Alechina et al., 2001). These are genuine discoveries that there are certain interesting and deep isomorphisms between modal logics and other first-class citizens of mathematics. Though by themselves they do not come pre-packaged with metaphysical, metalogical, conclusions about what sort of vast realm we should believe in. For example, knowing that a certain variety of topological (McKinsey & Tarski, 1944) or sheaf-semantics (Suzuki, 1999) will satisfy the axioms of **S4**—even assuming we are ourselves committed to **S4** for some reason—does not tell us that we should believe the realm of *possibilia* consists of things that are topology- or sheaf-like.^13^ There are lots of mathematical structures that validate the same modal axioms—a train set may satisfy the axioms of **S4**, under a chosen interpretation of stations as points with accessibility given by train routes. Nonetheless, if a category-like approach *could not* meet the fundamental needs of modal logic, that would be a significant mark against it. This section shows that even a naïve categorialization—one that allows quantifying over possible world-morphisms—can meet the needs of providing models for modal logics.

I will neglect the full description of a semantics in order to focus just on the essentials required to use a (pointed) category as a model of modal sentences. Consider a sentential language $\mathcal {L}$. An arrow theoretic model $\mathfrak {M}$ of $\mathcal {L}$ will consist of a collection of objects $obj(\mathfrak {M})$ and morphisms $arr(\mathfrak {M})$ with some specified object *w* (or its identity morphism $Id_{w}$, when available) chosen as actual. An arrow theoretic model is not assumed to satisfy the category axioms. In the background we require an assignment $\mathcal {V}: obj(\mathfrak {M}) \rightarrow {\textbf {V}}$ of propositional truth-value assignments ${\textbf {V}} \ni v_{i}: \mathcal {L} \rightarrow \{0,1\}$ to objects of the model. For brevity I will refer to the codomain of a function $f:x\rightarrow y$ with domain *x* simply as *f*(*x*). I will use ‘$\implies$’ as meta- and object-language conditional, since no confusion should result.

With these notions in hand, we can define $\phi$-modalities by $\phi$-preserving morphisms in a model, as follows. Assuming that the morphisms of $\mathfrak {M}$ are $\phi$-preserving,

1 $$\begin{aligned} \mathfrak {M} \models _{w} \square _{\phi } A \iff (\forall _{f})( dom(f)=w \implies \mathcal {V}(f(w)) \models A) \end{aligned}$$

And likewise,

2 $$ \begin{aligned} \mathfrak {M} \models _{w} \Diamond _{\phi } A \iff (\exists _{f})( dom(f)=w \ \& \ \mathcal {V}(f(w)) \models A) \end{aligned}$$

Ignoring the type of modality under consideration and defining $\mathfrak {M} \models _{f(w)} =_{df} \mathcal {V}(f(w)) \models$, this can be further simplified as,

3 $$\begin{aligned} \mathfrak {M} \models _{w} \square A \iff (\forall _{f})( dom(f)=w \implies \mathfrak {M} \models _{f(w)} A) \end{aligned}$$

And likewise,

4 $$ \begin{aligned} \mathfrak {M} \models _{w} \Diamond A \iff (\exists _{f})( dom(f)=w \ \& \ \mathfrak {M} \models _{f(w)} A) \end{aligned}$$

Plainly, arrow theoretic models allow us to express what we could within standard Kripke semantics. The objects $obj(\mathfrak {M})$ play the role of the set of worlds *W* and the arrows $arr(\mathfrak {M})$ define an accessibility relation *R* according to $\langle x,y \rangle \in R \subseteq W \times W \iff f: x \rightarrow y \in arr(\mathfrak {M})$. Doubtless other potentially interesting analogies between both approaches can be made. I concentrate on the relationship between types of categories and corresponding conditions on accessibility relations.

Firstly, I stress that the assumption that an arrow theoretic model is a fully fledged category allows the elimination of reference to possible worlds from the definition of the model and from the definition of truth in the model—by replacing *w* with $Id_{w}$ and ‘*f*(*w*)’ with ‘$f \circ Id_{w}$’—though it is slightly less convenient to do so. This conclusion does not come for free, since stipulating that $\mathfrak {M}$ is a category serves the same role as claiming that the frame $\langle W, R \rangle$ is reflexive and transitive. In other words,

#### Theorem 1

If $\mathfrak {M}$ is a category then it validates **S4**.

#### Proof

The assumption that $\mathfrak {M}$ is a category validates the axioms **T** $\square A \implies A$ and **4** $\square A \implies \square \square A$ of **S4**. This follows directly from the axioms of existence of identities and existence of composites, respectively. Supposing $\mathfrak {M} \models _{w} \square A$, by definition $(\forall _{f})( dom(f)=w \implies \mathfrak {M} \models _{f(w)} A)$. Since $dom(Id_{w}) = w$, the existence of such identities for each *w* gives $\mathfrak {M} \models _{Id_{w}(w)} A$, so $\mathfrak {M} \models _{w} A$, validating **T**. Likewise, supposing $\mathfrak {M} \models _{w} \square A$, by definition $(\forall _{f})( dom(f)=w \implies \mathfrak {M} \models _{f(w)} A)$. Now consider any *g* composable with any *f* as above. Since $\mathfrak {M}$ is a category $g \circ f$ exists for any composable pair. Since $dom(g \circ f) = dom(f) = w$, it is clear that $g \circ f$ also satisfies the above, so $\mathfrak {M} \models _{ g \circ f (w)} A$. So $(\forall g)(\forall f)((dom(f) = w \wedge dom(g)=f(w)) \implies \mathfrak {M} \models _{g(f(w))} A)$ since *g* arbitrary. This is classically equivalent to $(\forall _{f})( dom(f)=w \implies (\forall _{g})( dom(g)=f(w) \implies \mathfrak {M} \models _{g(f(w))} A))$. Using the definition of truth relative to a model once on the consequent, this is equivalent to $(\forall _{f})( dom(f)=w \implies \mathfrak {M} \models _{f(w)} \square A))$, which is the definition of $\mathfrak {M} \models _{w} \square \square A$, validating **4**. $\square$

Perhaps for some this would be a reason to prefer **S4**. To eliminate possible worlds from the definition we end up requiring enough structure to satisfy **S4**. To be general enough to model modal logics weaker than **S4** we could allow that $\mathfrak {M}$ be a “semicategory” or other weaker arrow theoretic construct. On the other hand, stronger systems can be obtained in similar fashion.

#### Theorem 2

If $\mathfrak {M}$ is a groupoid then it validates **S5**.

#### Proof

*Omitted* A groupoid is a category that has an inverse for every arrow. That is, its underlying relational structure is an equivalence relation, and equivalence relations validate **S5**. $\square$

Evidently the use of categorial models provides ready-made equivalents of familiar propositional modal notions and systems. This is enough for my main argument: categories are at least as good at underpinning propositional modal logic. However, using categories instead of relational structures as models of these familiar systems is overkill—on par with using a sledgehammer to crack a shell. This is not the way the founders of category theory justified their shift in perspective (see McLarty, 2003). To see that the use of categories as models may add something interesting to the existing practice of using Kripke models, I conclude my discussion of propositional modal logics by showing a more general result about the relationship between the semantics based on pointed categories and Kripke-models: the two are adjoint.

Kripke-models $\mathfrak {K} = \langle W, R \supseteq W \times W, w \in W \rangle$ are usually described as consisting of a set *W* of worlds, a relation *R* of accessibility between worlds and an actual world *w* selected from *W*. Dropping the metaphysical terminology, a Kripke-model is a “pointed related set”, a triple $\mathfrak {K} = \langle W, R, w \rangle$, consisting of a set *W*, a relation *R* on *W*, and a point $w \in W$. Kripke-models are the objects of the category ${\textbf {pRel}}$, whose morphisms are relation and point preserving maps (see Rydeheard & Burstall, 1988; Adámek et al., 2004; Brunet, 2021, p. 10901), i.e. a morphism $f: \langle W, R, w \rangle \rightarrow \langle W', R', w' \rangle$ is a function $f: W \rightarrow W'$ satisfying,

$$\begin{aligned} Rab&\Rightarrow R'f(a)f(b) \\ f(w)&= w' \end{aligned}$$

As characterized above, a categorial-model of a sentential language is just a “pointed category”, i.e. a pair $\langle \mathfrak {C}, c \rangle$ consisting of a category and some object of that category selected as the point, and so these categorial-models form the objects of a category ${\textbf {pCat}}$. The morphisms $F: \langle \mathfrak {C}, c \rangle \rightarrow \langle \mathfrak {C'}, c' \rangle$ of this category are just functors $F: \mathfrak {C} \rightarrow \mathfrak {C'}$ satisfying $F(c) = c'$.

We can now define two functors $U: {\textbf {pCat}} \leftrightarrows {\textbf {pRel}}: F$ where *U* is the forgetful functor from ${\textbf {pCat}}$ to ${\textbf {pRel}}$ that “forgets” all the categorial structure except the set $obj(obj({\textbf {pCat}}))$ and relational structure imposed by $arr(obj({\textbf {pCat}}))$, and *F* is a free-functor from ${\textbf {pRel}}$ to ${\textbf {pCat}}$ that maps to the free-category on a set generated by the relation. Where $\langle \mathfrak {C}, c \rangle$ is some pointed category,

$$\begin{aligned} U(\langle \mathfrak {C}, c \rangle )&= \langle obj(\mathfrak {C}), R_{\mathfrak {C}}, c \rangle \\ R_{\mathfrak {C}}&= \{ \langle a, b \rangle \ | \ \exists _{f \in arr(\mathfrak {C})} \ f: a \rightarrow b \} \end{aligned}$$

where $\mathfrak {K} = \langle W, R, w \rangle$ is some pointed related set, a Kripke-model,

$$\begin{aligned} F(\mathfrak {K} = \langle W, R, w \rangle )&= \langle \mathfrak {C}_{\mathfrak {K}}, w \rangle \\ obj(\mathfrak {C}_{\mathfrak {K}})&= W \\ arr(\mathfrak {C}_{\mathfrak {K}})&= \Delta (W) \cup \{ \langle w_{i},...w_{n} \rangle \ | \ Rw_{j}w_{j+1} \} \\ \Delta (W)&= \{ \langle w, w \rangle \ | \ w \in W\} \end{aligned}$$

That is, $arr(\mathfrak {C}_{\mathfrak {K}})$ consists of *R*-linear paths in $\mathfrak {M}$ together with the diagonal $\Delta (W)$. Composition of arrows is given by concatenation (joining tuples that overlap), and identity arrows are given in $\Delta (W)$.

*F* and *U* are adjoint and the adjunction is given just as it is for the familiar adjunction ${\textbf {Grph}} \rightharpoonup {\textbf {Cat}}$ (Mac Lane, 2013, p. 48, since graphs are essentially just related sets), while imposing conditions for preservation of pointness as in the adjunction ${\textbf {Set}} \rightharpoonup {\textbf {pSet}}$. It remains just to see that *UF* is the identity on the collection *W* “of worlds”, the identity on *w* the “actual world” and the transitive-reflexive closure on the “accessibility” relation *R*. That is, if we take a given Kripke model, construct the (pointed) free category on the relation *R* of its frame, then forget the categorial structure to give the underlying (pointed) relation of this category, then the relation we obtain will be relextive and transitive. This immediately gives the following result.

#### Theorem 3

For every Kripke-model $\mathfrak {K}$ the underlying Kripke-model of the free-pointed-category generated by $\mathfrak {K}$, i.e., $UF\mathfrak {K}$, validates ${\textbf {S4}}$.

Showing that Kripke-models and categorial-models are related by a pair of opposing (adjoint) functors is enough to submerge them in the fundamental notions of category theory.

I now turn to quantified modal logic (QLM) and models using counterpart functors. Consider a first order modal language $\mathcal {L}^{1}$, consisting of $\mathcal {L}^{1}_{CONS}$ the constants of the language, $\mathcal {L}^{1}_{FUNC}$ function symbols, $\mathcal {L}^{1}_{VARS}$ variables, and $\mathcal {L}^{1}_{PRED}$ predicates. Among the predicates we will have a distinguished trinary predicate symbol ‘$\_: \_ \rightarrow \_$’ with the intended interpretation of stating the codomain (third place) and domain (second place) of a morphism (first place). We will also have a distinguished binary symbol ‘$\circ$’ with the intended interpretation of being the (partially defined) composition of morphisms. The language $\mathcal {L}^{1}$ will also include ‘$\forall$’, ‘$\exists$’, ‘$\square$’, ‘$\Diamond$’ and the usual propositional connectives.

I will provide a model of a single dual pair of modalities for a single sort of $\phi$-counterpart, though the generalization to a multiplicity of counterpart functors is straightforward. A $\phi$-counterpart functor model $\mathfrak {M}^{1}$ for the language $\mathcal {L}^{1}$ of QLM will be defined as a 5-tuple.

5 $$\begin{aligned} \mathfrak {M}^{1} = \langle obj(\mathfrak {M}^{1}), arr(\mathfrak {M}^{1}), I_{()}, F, \mathfrak {w} \rangle \end{aligned}$$

here $obj(\mathfrak {M}^{1})$ is the collection of worlds, such that each $\mathfrak {a} \in obj(\mathfrak {M}^{1})$ is itself arrow theoretic, i.e., $obj(\mathfrak {a})$ and $arr(\mathfrak {a})$ are defined collections. Accessibility is again given by world morphisms $\mathcal {F}: \mathfrak {a} \rightarrow \mathfrak {b} \in arr(\mathfrak {M}^{1})$, where $\mathcal {F}$ may be considered as a pair of morphisms $\mathcal {F}: obj(\mathfrak {a}) \rightarrow obj(\mathfrak {b})$ and $\mathcal {F}: arr(\mathfrak {a}) \rightarrow arr(\mathfrak {b})$. The local interpretation $I_{()}: obj(\mathfrak {M}^{1}) \rightarrow \mathcal {I}$ gives interpretation functions $I_{\mathfrak {a}} \in \mathcal {I}$ for each world $\mathfrak {a} \in obj(\mathfrak {M}^{1})$, defined on the language by giving objects, arrows, or subsets of products of $\mathfrak {a}$,

$$\begin{aligned}&I_{\mathfrak {a}}: \mathcal {L}^{1}_{CONS} \rightarrow obj(\mathfrak {a}) \\&I_{\mathfrak {a}}: \mathcal {L}^{1}_{FUNC} \rightarrow arr(\mathfrak {a}) \\&I_{\mathfrak {a}}: \mathcal {L}^{1}_{PRED} \rightarrow \mathcal {P}(obj/arr(\mathfrak {a})^{n}) \\ \end{aligned}$$

where $obj/arr(\mathfrak {a})^{n}$ is all the n-tuples of either objects or arrows of $\mathfrak {a}$ for any n. The collection of $\phi$-counterpart functors *F* is defined as follows. For each $\mathcal {F}: \mathfrak {a} \rightarrow \mathfrak {b} \in arr(\mathfrak {M}^{1})$, *F* contains the restriction $\hat{\mathcal {F}}: \mathfrak {a}|_{\phi } \rightarrow \mathfrak {b}$ of $\mathcal {F}$ to the subworld $\mathfrak {a}|_{\phi }$ on which $\mathcal {F}$ preserves $\phi$, as in the diagram below.^14^
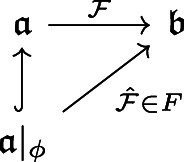


This defines the frame. Finally, to provide a model we must select some $\mathfrak {w} \in obj(\mathfrak {W}^{1})$ as the actual world.^15^

The truth conditions for sentences of $\mathcal {L}^{1}$ can now be provided. For the first order cases, the truth conditions are given by treating $\langle obj(\mathfrak {a}) \cup arr(\mathfrak {a}), I_{\mathfrak {a}} \rangle$ as a standard first order model structure, for each $\mathfrak {a}$. Where *P* is some n-ary predicate of $\mathcal {L}^{1}$, $\bar{c}$ an n-ary sequence of constants, *f* a function symbol, *s*[*x*/*y*] a satisfaction function which differs from *s* at most by assigning *y* to *x*, and $w \in \mathfrak {w}$

$$ \begin{aligned} \mathfrak {M}^{1} \models _{\mathfrak {w}} P\bar{c}&\iff I_{\mathfrak {w}}(\bar{c}) \in I_{\mathfrak {w}}(P) \\ \mathfrak {M}^{1} \models _{\mathfrak {w}} f:c \rightarrow c'&\iff I_{\mathfrak {w}}(f): I_{\mathfrak {w}}(c) \rightarrow I_{\mathfrak {w}}(c') \\ \mathfrak {M}^{1} \models _{\mathfrak {w}} f \circ g = h&\iff I_{\mathfrak {w}}(f) \circ I_{\mathfrak {w}}(g) = I_{\mathfrak {w}}(h) \\ \mathfrak {M}^{1} \models _{\mathfrak {w}} \psi \wedge \chi&\iff \mathfrak {M}^{1} \models _{\mathfrak {w}} \psi \ \& \ \mathfrak {M}^{1} \models _{\mathfrak {w}} \chi \\ \mathfrak {M}^{1} \models _{\mathfrak {w}} \lnot \psi&\iff \mathfrak {M}^{1} \not \models _{\mathfrak {w}} \psi \\ \mathfrak {M}^{1} \models _{\mathfrak {w}} (\forall x) \psi x&\iff \mathfrak {M}^{1} \models _{\mathfrak {w},s[x/w]} \psi x \text { for all s, for all w} \in \mathfrak {a} \end{aligned}$$

For the modal cases, truth will be defined by quantification over worlds and world-morphisms, and will depend on the presence of individuals in the domain of the associated counterpart functor. Considering only monadic *P* and some constant *c* for convenience, $I_{\mathfrak {w}}(c) \in dom(\hat{\mathcal {F}})$ is the special clause specifying that *c* has such-and-such a counterpart (defined by $\hat{\mathcal {F}}$) according to the model.

$$  \begin{aligned}&\mathfrak {M}^{1} \models _{\mathfrak {w}} \Diamond Pc \iff (\exists \mathfrak {w}')(\exists \mathcal {F}) ( \mathcal {F}: \mathfrak {w} \rightarrow \mathfrak {w}' \, \& \, I_{\mathfrak {w}}(c) \in dom(\hat{\mathcal {F}})\, \& \,\hat{\mathcal {F}}(I_{\mathfrak {w}}(c)) \in I_{\mathfrak {w}'}(P)) \\&\mathfrak {M}^{1} \models _{\mathfrak {w}} \square Pc \iff (\forall \mathfrak {w}')(\forall \mathcal {F}) (( \mathcal {F}: \mathfrak {w} \rightarrow \mathfrak {w}'\, \& I_{\mathfrak {w}}(c) \in dom(\hat{\mathcal {F}})) \implies \hat{\mathcal {F}}(I_{\mathfrak {w}}(c)) \in I_{\mathfrak {w}'}(P)) \end{aligned}$$

That is, it is possible that *Pc* at $\mathfrak {w}$ iff there is a world $\mathfrak {w}'$ and world morphism $\mathcal {F}$ such that, the morphism makes the world accessible $\mathcal {F}: \mathfrak {w} \rightarrow \mathfrak {w}'$, the restriction of that morphism to the sort of counterparthood under consideration $\hat{\mathcal {F}}$ is defined on the interpretation of *c* at $\mathfrak {a}$, and the counterpart of *c* according to the world morphism is an element of the interpretation of *P* at $\mathfrak {w}'$. Dually for necessity. Validity is defined by quantifying over the world of evaluation, as usual.

I neglect a full investigation of the relationships between conditions on such models and modal principles. However, we can see how some such relationships can be established by considering how the above models relate to typical models of QML with variable domains. A set theoretic model of QML (see e.g. Corsi, 2002, p. 10) is a 6-tuple: $\mathfrak {S} = \langle W, R, D_{()}, C, I^{\mathfrak {S}}, w \rangle$ where *W* is a set (of worlds), *R* a relation (of accessbility), $D_{()}$ is a function from worlds *w* to domains of those worlds $D_{w}$, *C* is a collection of counterpart relations $C_{w,w'}$ for each $w,w' \in W$, $I^{\mathfrak {S}}$ is a function giving (local) interpretations for each world, and $w \in W$ is the actual world.

The set theoretic and categorial models above are related in the following way—allowing us to view the set theoretic models as the discrete case of the categorial models. Every $\phi$-counterpart functor model $\mathfrak {M}$ gives rise to a set theoretic model $\mathfrak {S_{\mathfrak {M}}}$ as follows. The set/class of worlds is given by the collection of objects of the model $W = obj(\mathfrak {M})$, the relation by the arrows of the model $Rww' \iff \exists \mathcal {F}: w \rightarrow w' \in arr(\mathfrak {M})$, the domain of each world is given by the objects and arrows of the worlds $D_{w} = obj(w) \cup arr(w)$, the counterpart relation is given by the counterpart functor $C_{w,w'} = \{ \langle x,y \rangle | y = \hat{\mathcal {F}}(x) \text { for all } \mathcal {F}: w \rightarrow w' \in arr(\mathfrak {M}) \}$. The interpretation $I^{\mathfrak {S}_{\mathfrak {M}}}$ and actual world are unchanged.^16^

This makes it easier to see how familiar modal principles relate to these functorial models, under limiting assumptions about their structure. For example,

#### Theorem 4

(The Barcan Formula) $\mathfrak {M} \models \forall x \square Fx \implies \square \forall x Fx$ iff $\hat{\mathcal {F}}$ is surjective on objects and morphisms, for all $\mathcal {F} \in arr(\mathfrak {M})$.

#### Proof

The result is essentially already proved (see Corsi, 2002, p. 29, Lemma 2.4). We need only note that, if $\hat{\mathcal {F}}$ is surjective on objects and morphisms, then its underlying relation is also surjective on the union of its objects and morphisms. $\square$

This is enough to establish my central claim: these sorts of models indeed provide a basis for quantified modal logic, at least as well as set theoretic models do. However, they also have something to add. I conclude this section with two observations specific to the categorial models introduced here.

First, the assumption that worlds are categories and that counterparts are given functorially implies that morphisms necessarily have their domains and codomains if only they exist at a world (as argued for informally at the end of § 3).

#### Theorem 5

$\mathfrak {M} \models f: a \rightarrow b \implies \square ((\exists x) f = x \implies f: a \rightarrow b)$ iff $\mathcal {F}: \mathfrak {a} \rightarrow \mathfrak {b}$ is a functor, for all $\mathfrak {a,b} \in \mathfrak {M}$

#### Proof

Assume $\mathfrak {M} \models _{\mathfrak {w}} f: a \rightarrow b$. By definition, $I_{\mathfrak {w}}(f): I_{\mathfrak {w}}(a) \rightarrow I_{\mathfrak {w}}(b)$. Now consider any $\mathfrak {b}$ such that $\mathcal {F}: \mathfrak {w} \rightarrow \mathfrak {b}$, and assume there is some $b_{f} \in arr(\mathfrak {b})$ such that $\hat{\mathcal {F}}(I_{\mathfrak {w}}(f)) = b_{f}$. Since $\mathcal {F}$ is a functor, $b_{f} = \hat{\mathcal {F}}(I_{\mathfrak {w}}(f)): \hat{\mathcal {F}}(I_{\mathfrak {w}}(a)) \rightarrow \hat{\mathcal {F}}(I_{\mathfrak {w}}(b))$. So $\mathfrak {M} \models _{\mathfrak {b}} (\exists x) f = x \implies f: a \rightarrow b$, but $\mathfrak {b}$ was arbitrary, so $\mathfrak {M} \models _{\mathfrak {w}} f: a \rightarrow b \implies \square ((\exists x) f = x \implies f: a \rightarrow b)$, but $\mathfrak {w}$ arbitrary, so the sentence is a validity. $\square$

Finally, these $\phi$-counterpart functor models can be used as natural models of a whole class of principles unique to languages as expressive as $\mathcal {L}^{1}$. Due to the inclusion of a distinguished predicate for morphisms and composition, with fixed interpretations, $\mathcal {L}^{1}$ is essentially a first-order language sufficient to express elementary^17^ properties of categories, enriched with a supply of other predicates and modals. For example, there is a first-order sentence of $\mathcal {L}^{1}$ stating any of the elementary *universal properties*, such as those for *products, coproducts, power-objects, etc.* That is, our language suffices to express sentences such as $\phi ^{\times }_{a \prod b, a,b,p_{1},p_{2}}$, for “$a \prod b$ is the product of *a* and *b* with projections $p_{1}$ and $p_{2}$”. Moreover, in the semantics, we can take any such universal property $\phi$ to determine a restriction on the class of (counterpart) functors admissible in a categorial model: the class of (universal) $\phi$-preserving functors. For example,

#### Theorem 6

$\mathfrak {M} \models \phi ^{\times }_{a \prod b, a,b,p_{1},p_{2}} \implies \square \phi ^{\times }_{a \prod b, a,b,p_{1},p_{2}}$ iff $\mathfrak {M}$ is a product-counterpart functor model.

#### Proof

Follows from the definition of $\phi ^{\times }$ and product preservation. If $\mathfrak {M}$ is a product-counterpart functor model, then $\hat{\mathcal {F}}$ preserves products, so must take $a \prod b$ to a counterpart that also satisfies $\phi ^{\times }$ relative to the counterparts of $a,b, p_{1},p_{2}$. Moreover, this will be true for all morphisms $\mathcal {F}$. $\square$

This gives a class of relationships between constraints on categorial models and modal formulas about universal properties. This theoretical option is rendered visible by the shift to a categorial view of the plurality and by the use of corresponding categorial models.

### Size: on the many ways to be many

One sort of objection to Lewis’ modal realism pertains to the size of the plurality of worlds.^18^ These objections typically rely on some axiomatic principle, either of modal logic or of Lewis’ view, to say that the plurality suffers from some “paradox akin to those that refute naïve set theory” (Lewis 1986, p. 101). Lewis addresses those of Forrest and Armstrong (1984), who provide a typical form of this objection. The objection latches onto some plausible version of the *principle of recombination* “according to which patching together parts of different possible worlds yields another possible world” (Lewis 1986, p. 87) and derives paradoxes of Russell’s variety by analogy with the principle of *unrestricted* comprehension in naïve set theory. Lewis (1986) characterizes the first part of the *reductio* as follows,

Start with all the possible worlds. Each one of them is a possible individual. Apply the unqualified principle of recombination to this class of possible individuals. Then we have one big world which contains duplicates of all our original worlds as non-overlapping parts. But we started with all the worlds; *so our big world must have been one of them. Then our big world is bigger than itself; but no matter how big it is, it cannot be that.— Lewis (1986, p. 102)

Lewis’ response is that his principle of recombination is not unrestricted in a way that leads to paradox—he suggests constraints on shape or size of spacetime—and reflective equilibrium naturally shifts focus back to whether a *restricted* principle of recombination is plausible. In Lewis’ response “size” was understood in terms of the number and cardinality of spatial dimensions; the response I argue for here uses a categorial conception of a relative size distinction of a plurality (a “small” vs. “large” distinction) on the basis of a given plurality and a notion of isomorphism of worlds. This resolves the size based objection to Lewis’ plurality by blocking the construction of a paradoxically large world, in a way that still allows for very “large” worlds—and does so without seemingly arbitrary constraints on the shape or size of possible spaces. Moreover, this approach relies on the idea that the categorial notion of *isomorphism* is fundamental to issues of the size or quantity of collections.^19^

This, perhaps more than any other, is an arena where the analogies between the plurality of worlds and the hierarchy of sets play a significant role. Lewis’ response is reasonable, a restricted principle does not suffer the proposed paradoxes, but it would be just as reasonable a response were the criticism lodged against the elementary theory of sets and classes.^20^ So, perhaps Lewis should have begun by analogy between the plurality of worlds and the theory of sets with proper classes.^21^ Lewis does not do this, not even retroactively. He is insistent that there is a *set* of worlds (1986, p.104) and provides a lower bound on the cardinality of this set as $\beth _{2}$ (Lewis, 2013, p. 90). The point of this section is that there is another option.

Consider Eilenberg and Mac Lane (1945, p. 246) on foundations,

[S]uch examples as the “category of *all* sets,” the “category of *all* groups” are illegitimate. The difficulties and antinomies here involved are exactly those of ordinary intuitive *Mengenlehre* [naive set theory]; no essentially new paradoxes are apparently involved. Any rigorous foundation capable of supporting the ordinary theory of classes would equally well support our theory. Hence we have chosen to adopt the intuitive standpoint, leaving the reader free to inset whatever type of logical foundation (or absence thereof) he may prefer. —Eilenberg and Mac Lane (1945)

The issue for Eilenberg and Mac Lane is whether the objects and morphisms of a category are sets. Provided *‘all’* is read unrestrictedly, this would mean that the category of *all* sets (or groups) would be inadmissible. That would be unfortunate, so substitute another notion when referring to the objects and morphisms collectively. It is common to say that a category consists of two ‘*classes*’, ‘*collections*’ or ‘*aggregates*’, where the impetus is just to interpret these equally foundational terms in *some* way that does not allow for the known paradoxes of size. There are problems with the “set of all sets” that there are not, for example, with the “class of all sets” or “collection of all sets”. The intuitive standpoint leaves off at this point. Evidently, if Lewis rejects the idea that the plurality of worlds is a proper class, then some other rigorous foundation is required. There are other ways to go about avoiding paradox while allowing for vast realms of entities suitable to category theory. I describe them here and suggest analogies in service of vast realms of possibility.

When the need arises to distinguish between *small* and *large* types of collections, the distinction between sets and proper classes often furnishes what is necessary. Some such distinction in hand, it becomes possible to distinguish between, for example, *small categories* and *large categories*—so that a small category can be defined as one where the *collections of objects and morphisms* are isomorphic to a *set* (Mac Lane, 1969). Paradox is avoided by defining smallness so that the “category of small categories” is not (necessarily) small.

By analogy, we *could* avoid paradoxes of size for the plurality of worlds by making a distinction between *small worlds* and *large worlds*. We would then define small worlds as those where the collection of individuals and morphisms form a set (or are set-like in some rich way). Then the collection of small worlds could be defined by closure under set operations or under some Lewis (1986) style principle of recombination. We can even *without paradox* form a ‘world formed by Lewis-recombination of all small worlds’, although that world could not itself be small. Here ‘small’ and ‘large’ serve by restricting quantification over worlds, so that we do not encounter the problem of “all worlds in one”, but only (the not self-evidently contradictory) “all small worlds in one large world”. This option is only available to us if we reject the idea that the plurality of worlds is a set, since it must be a class for this approach to work. If, like Lewis, we also reject that it is a proper class, then we require some other foundation.

Another option is to choose a particular *Grothendieck universe* $\mathfrak {U}$ according to which one defines smallness of a world *w* via isomorphisms $w \cong x$ with elements $x \in \mathfrak {U}$ (Artin et al., 1973). Note, importantly, that we do not need to construe the element relation ‘$\in$’ set theoretically (Goldblatt, 1981, Chap. 3; Lawvere, 1966). Provided $\mathfrak {U}$ satisfies certain conditions it can serve a similar role in marking size distinctions, while being more flexible than the binary distinction between sets and proper classes. The axioms for Grothendieck universes are as follows,

- $\mathfrak {U}$ 0 $\mathfrak {U}$ is non-empty,
- $\mathfrak {U}$ 1 if $x \in \mathfrak {U}$ and $y \in x$, then $y \in \mathfrak {U}$.
- $\mathfrak {U}$ 2 for any pair of elements $x,y \in \mathfrak {U}$ there is a set $\{x,y \} \in \mathfrak {U}$.
- $$\mathfrak {U}$$ 3 if $$x \in \mathfrak {U}$$ then $$\mathcal {P}(x) \in \mathfrak {U}$$.
- $\mathfrak {U}$ 4 if $(x_{i}, i \in I) \in \mathfrak {U}$ is an indexed family of element of $\mathfrak {U}$ and $I \in \mathfrak {U}$, then $\cup _{i \in I} x_{i} \in \mathfrak {U}$ (the union of families of elements of $\mathfrak {U}$ that are indexed by elements of $\mathfrak {U}$ are themselves elements of $\mathfrak {U}$).

Relevant for us $\mathfrak {U} \in \mathfrak {U}$ is not derivable from $\mathfrak {U}0- \mathfrak {U}4$. This allows us to go about performing set-operations as usual, within a particular universe.^22^ Moreover, if we wish to permit ourselves sets of any cardinality, we can append an additional *axiom of universes* (referred to as $\mathfrak {U}A$ in Artin et al. 1973),

($\mathfrak {U}A$) For every set *x* there exists a universe $\mathfrak {U}$ such that $x \in \mathfrak {U}$.

The connection to comparative measures of size is given by defining a set (or other algebraic object) as $\mathfrak {U}$-small (or “little”) if it is *isomorphic* to an element of $\mathfrak {U}$.^23^ Finally, it is no matter that the collection of $\mathfrak {U}$-small sets is not $\mathfrak {U}$-small, since by $(\mathfrak {U}A)$ we can assert the existence of some other “larger” $\mathfrak {U'}$, of which it is an element and relative to which it is $\mathfrak {U}'$-small.

By analogy, in service of a vast realm of paradox free possibility, assume some particular universe of possibilities $\mathfrak {W}$. The aim would then be to define that universe according to suitable mereological analogues of the axioms for a Grothendieck universe $\mathfrak {U}$. My suggestion is that $\mathfrak {W}$ should have following directly analogous properties,

- $\mathfrak {W}$ 1 If $x \in \mathfrak {W}$ and *y* is a part of *x*, then $w_{\{y\}} \in \mathfrak {W}$, for $w_{\{y\}}$ a world containing only an intrinsic duplicate of *y*.
- $\mathfrak {W}$ 2 If $x, y \in \mathfrak {W}$ then there is a world $w_{\{x,y\}} \in \mathfrak {W}$, where $w_{\{x,y\}}$ is obtained by “patching together” the worlds *x* and *y* as parts within a single world.
- $\mathfrak {W}$ 3 If $x \in \mathfrak {W}$ then $w_{\mathcal {P}(x)} \in \mathfrak {W}$, where $w_{\mathcal {P}(x)}$ is a world obtained by “patching together” all of the worlds $w_{y}$ where *y* is a part of *x*.
- $\mathfrak {W}$ 4 If $I \in \mathfrak {W}$ and $\{x_{i}\}_{i \in I} \in \mathfrak {W}$ is a family of $\mathfrak {W}$ worlds indexed by $i \in I$, then $w_{\bigcup _{i \in I} x_{i}} \in \mathfrak {W}$, where $w_{\bigcup _{i \in I} x_{i}}$ is a world formed by “patching together” all the worlds indexed by *I*.

Of course, there is necessary ambiguity about how worlds are patched together and how parthood works within worlds, but this ambiguity is beside the point (and already in Lewis, 1986). The point is just that—provided suitable disambiguations—from these axioms we can easily define notions analogous to those in Artin et al. (1973). For the present connection, this is just enough to say that the elements of $\mathfrak {W}$ are smaller than it and, in particular, that $\mathfrak {W} \in \mathfrak {W}$ does not obtain. We likewise obtain another workable connection to size by defining a world as $\mathfrak {W}$-small if it is *isomorphic* to an element of $\mathfrak {W}$. On this approach, considerations of world-size and allowable compositions of worlds turn on the existence of world-isomorphisms $\theta : w \cong w' \in \mathfrak {W}$. Moreover, if we wanted to allow the existence of worlds of any size, we should append an analogue $(\mathfrak {W} A)$ of the axiom $(\mathfrak {U} A)$.

($\mathfrak {W} A$) For every world *x* there exists a plurality $\mathfrak {W}$ such that $x \in \mathfrak {W}$.

Then, we could without paradox assert the existence of the world formed by patching together all the $\mathfrak {W}$-small worlds, itself within some larger $\mathfrak {W'}$.

We can now see how this categorial approach affects the arguments against modal realism, offered by Forrest and Armstrong (1984), as Lewis characterizes them. Notice that the *reductio* can be blocked (at the * in the first quote from Lewis in this section) provided we include notions of comparative size, assuming the requisite isomorphisms from the very beginning. This would look as follows: Consider some universe of *possibilia* $\mathfrak {W}$. Start with all the $\mathfrak {W}$-small possible worlds. Each one of them is a possible $\mathfrak {W}$-small part of a world. Apply the unqualified principle of recombination to this class of possible $\mathfrak {W}$-small parts. Then we have one $\mathfrak {W}$-large world which contains duplicates of all our original worlds as non-overlapping parts. But since we started with all the $\mathfrak {W}$-small worlds; our $\mathfrak {W}$-large world must *not* have been one of them.

With this categorial framework in hand we have some more choices of foundation. One option now available to us is to multiply notions of plurality under consideration.

Perhaps the simplest precise device would be to speak not of *the* category of groups, but of *a* category of groups (meaning any legitimate such category).—Eilenberg and Mac Lane (1945, p. 247)

By analogy, we would cease referring to *the* plurality of worlds, instead always speaking of *a* plurality of worlds (meaning some legitimate such plurality). For example, we could amend our talk of a set of all possible worlds or set of all *possibilia* to engage only with realism about the $\mathfrak {W}$-large plurality of $\mathfrak {W}$-small worlds.^24^

If we begin to allow worlds of any isomorphism class, how does this affect our comparison of the categorial and set-like ontology? One of the remarkable things about Lewis’ vast plurality of worlds is that he adopted it without giving up on Quine’s taste for desert landscapes, for simplicity as a theoretical virtue (see Janssen-Lauret 2017).

Our acceptance of an ontology is, I think, similar in principle to our acceptance of a scientific theory, say a system of physics : we adopt, at least in so far as we are reasonable, the simplest conceptual scheme into which the disordered fragments of raw experience can be fitted and arranged.—Quine (1948, pp. 35–36)

The plurality is a vastly populated universe, but it is not, on Lewis’ view, overpopulated. On the contrary, Lewis thinks it is the smallest ontology that will still do the job of a metaphysics of modality. To argue for this, Lewis needed only to adopt Quine’s standard for assessing the ontological commitments of a theory and show that his modal realism was preferable to theories that quantified over less.

This is done by arguments against ersatz metaphysics of modality. However, Lewis never confronts the problem of adjudicating between his modal realism and a realism with a similarly-sized ontology, a coequally desertified landscape, since he rejects all the other metaphysics of modality on offer. Lewis’ rejection of ersatzisms puts him in the trivial position of acceptance of an ontology as the simplest because it is *sui generis*, into no other ontology can the disordered fragments of philosophy be fit and arranged, on his view. The categorial ontology for modal realism advocated in this paper at very least remedies that triviality by providing another non-ersatz ontology for comparison. Lewis’ set-like plurality is not the only contender, so not trivially lightweight.

It is not clear to me which ontology is more “simple”, nor that “simplicity” should be the criterion of ontology choice. On the former, as Quine himself notes, simplicity is “not a clear and unambiguous idea” (p. 36). ‘Simplicity’ encompasses a series of closely related ideas, such as parsimony of assumptions or axioms, having fewer primitive notions or terms, and ease of use or inference within the system. And on the latter, that simplicity should be the reigning criterion of theory choice is not Quine’s view, nor should it be ours. We have added many theoretical virtues to the docket when adjudicating between theories. For instance, we might adjudicate on the basis of Kuhn’s (1970) five theoretical virtues—accuracy, consistency, scope (unification), simplicity, and fruitfulness—or on some expanded list (see Keas, 2018). On scope, unification and fruitfulness the category theoretic approach to mathematics can boast a high score (Marquis, 2021). Indeed, likewise on parsimony of axioms. The category axioms together with the axioms for Grothendieck universes together number less than the axioms of ZFC.

## Conclusion: quinean humility

I advanced an explicit standard whereby to decide what the ontological commitments of a theory are. But the question of what ontology actually to adopt still stands open, and the obvious council is tolerance and an experimental spirit.—Quine (1948, p. 38)

To conclude my argument for a categorial modal realism I recommend an epistemological take on the justification for belief in vast realms of possibility. I recommend a form of epistemological humility that, I think, is true to Quine’s stance on adoption of belief in the ontological commitments of a theory. I argue that this supports tolerance of categorial modal realism.

Quine famously provided a way to decide on the ontological commitments of a theory on the basis of the referents of the (quantified) variables of the theory. What are we to do when there is no unique class of referents which satisfy our theory? Quine, in another context, provides an answer. Quine’s (1968, pp. 197–198) *Ontological Relativity* deals with the problem of what to say about numbers in the theory of arithmetic, given that there are intrinsically distinct ways of making number terms refer to sets while keeping the theory, structure, of arithmetic intact—that is, there is no unique class of referents which satisfy the theory of arithmetic. His conclusion: “there is no saying absolutely what numbers are” (p. 198). Connecting this to his view of theory choice gives us strong reasons to be humble about our ontological commitments when a theory does not uniquely determine its satisfiers.

Langton (1998) advocated for a view called Kantian Humility, followed by Lewis’ (2001) Ramseyan Humility (contrasted in Langton 2004). Both are forms of scepticism restricted to knowledge about fundamental things. Kantian Humility is scepticism about knowledge of the intrinsic nature of substances; Ramseyan Humility is scepticism about the perfectly natural properties of the fundamental realizers of our theories. These are different views, however, as Langton (2004, p. 132) notes, “[i]n both we have the key ideas that *there are intrinsic properties*, and that *we do not know them*.” The arguments for these positions are also similar in the following way: our knowledge about things, or evidence for our theories, is obtained by being in a given relation to things, i.e. by relational properties, and it is possible for the intrinsic properties of things to change while their relational properties remain the same. This gives us no reason to believe in some *particular* nature to the intrinsic properties on the basis of our best theory. There are empirically equivalent theories with identical extrinsic relations and distinct intrinsic properties, so we should be humble about the particular nature of the intrinsics. This leads to scepticism about intrinsic properties in a Quinean way.

Janssen-Lauret and Macbride (2020 see their § 4–5) have argued that Lewis’ Ramseyan Humility is conceptually and historically derived from consideration of Quinean structuralism. Even if we assume we have some complete and final theory T, and we have evidence that some objects satisfy T, we still do not know *which* objects satisfy it, since our only knowledge of those objects is as satisfiers of T—“our only knowledge of them is knowledge of them qua theoretical role-fillers” [ibid, p. 21]—just as, on Quine’s mathematical structrualism, our only knowledge of number concepts is as (any of the) satisfiers of the laws of arithmetic. This gives us a Quinean Humility: when there is not a unique collection of entities that satisfy our best theories we do not know what the ontological commitments of our theory are, there is no saying absolutely what those commitments should be, so we should remain humble about which ontology to adopt and tolerant of any ontology that is one of the satisfiers of our theory.

I think Quinean Humility together with the plausibility of a categorial ontology justify a slightly more extreme scepticism about intrinsic properties: *it is possible to have an ontology where there are no intrinsic properties whatever* (and even if there are, as with Kantian and Ramseyan Humility, we do not know which ones there are). One of the growing fruits of categorial approaches to traditionally set-theoretical topics is the use of entirely structural or relational theories (see McLarty, 1993; Awodey, 1996; McLarty, 2004). Barring some fundamental problem with this approach, it is at least plausible to be a realist only about structural or extrinsic properties of our theories, whether these theories are scientific (see Bain, 2013; c.f. Lam & Würthrich, 2015) or metaphysical. A vast realm of individuals with intrinsic properties has indeed been a paradise for naturalistic philosophers, but it is not the only one. A vast structure of morphisms with extrinsic properties is also a paradise. As I argued above, the assumption that interesting ontological categories satisfy the category axioms comes with a host of theoretical benefits. Chief of which is that it becomes possible to define many of the theories we are interested in structurally: without reference to objects and their intrinsic properties. This is an even stronger reason that we could not know what the intrinsic properties are.

This (Quinean) structuralism about the referents of our theories is usually proposed for theories of natural science or mathematics. However, the same sort of reasoning applies to metaphysical theories. In particular, that there are two plausible satisfiers of our theory of alethic modality implies that we should be Quineanly humble about the ontological commitments of our theory of modality. Lewis’s set-like plurality of worlds is a satisfier of our best theory of modality where the referents are possible individuals forming a set, but it is not the only satisfier. A categorial account of the plurality is also a satisfier of our best theory of modality, one where the referents are possible morphisms forming a category. Since there are two, we should be humble about which of these ontologies we are committed to and tolerant of the other. In particular, I have argued that we should be tolerant of the central idea that possibility claims can be grounded entirely in collections of possible morphisms. If so, then there is no saying absolutely what possibilities are.

## Footnotes

1 There is a class of categories (see Lawvere & McLarty, 2005), the *discrete categories* (i.e. those where all morphisms are identities), that are isomorphic to sets (classes) provided only that they have a set (class) of objects. I argue below that it is helpful to think of the collection of worlds as a category, though surely discrete categories contribute nothing that is not isomorphically contributed by sets.

2 See Mac Lane (2013), Lawvere and Schanuel (2009), Awodey (2010).

3 This is actually a contentious point. Categories are often defined by explicit reference to collections of objects and collections of morphisms, but all categorial notions *can* be defined without reference to a collection of objects (discussed below). It is also contentious whether it is appropriate to treat the collection of objects as a *set* of objects (Sect. 4.2.) or as some other sort of collection.

4 That is, a function $f: G \rightarrow H$ between groups $(G,+)$ and $(H, *)$, must satisfy $f(x+y) = f(x)*f(y)$ for all $x,y \in G$ to be a group-homomorphism.

5 A set $\langle A, a \in A \rangle$ is pointed when equipped with an element $a \in A$ from the set *A* selected as the “point”.

6 It is typical to regard these (both) as morphisms of **Set** the category of sets, but it is not essential to do so.

7 In the following sections we will need more. Specifically we will require the notion of isomorphism and adjunction (see Mac Lane, 2013, pp. 19, 79).

8 I do not think that anything significant turns on ‘category’ being used in an ontological context while taken from one mathematical, nor that we are at risk of harmful equivocation. The important point is just that some changes (or processes) preserve the properties required to be of a given category and just these are to count as morphisms. This is a helpful repossession of the idea of a category for philosophical ontology, after it was borrowed and greatly generalized by mathematicians. Mac Lane (2013, pp. 29–30) says, “[T]he discovery of ideas as general as these is chiefly the willingness to make a brash or speculative abstraction, in this case supported by the pleasure of purloining words from the philosophers: “Category" from Aristotle and Kant, “Functor” from Carnap...”. Moreover, for their part, mathematicians often draw similar analogies between the mathematical sense of ‘function’ and physical changes, e.g., Lawvere and Schanuel (2009) refer to functions as processes.

9 Here, the world $w'$ is serving analogously to an arrow category $\mathfrak {C}^{\rightarrow }$.

10 Some world including the actual world. On a processualist account, it is appropriate (in the actual world) to treat life-cycles and species as individual processes (Dupré, 2017; Nicholson & Dupré, 2018).

11 An organism-identity preserving morphism between individuals with a high-sugar diet.

12 An organism-identity preserving morphism between individuals without a high-sugar diet.

13 c.f. Brunet (2021). Imposing a categorial sheaf structure on models of the plurality of worlds has other advantages, such as providing a local analysis of causation.

14 The object $\mathfrak {a}|_{\phi }$ need not be a world in the model, and $\phi$ need not be a property expressible in the language $\mathcal {L}^{1}$—they just serve to capture the special clause, specifying some sort of counterpart regarded as such-and-such ($= \phi$).

15 Excluding the assumption that each world itself is an arrow theoretic object, the models defined here are structurally similar to those of Corsi (2002, p. 10) and Ghilardi and Meloni (1988, p. 131). The reader is encouraged to see Ghilardi and Meloni’s (1988, p. 135) “informal interpretation” of their (categorial) “universes for tense predicate logic”. Specifically, note their description of arrows within their model as “possible temporal developments [of worlds]”, as well as their description (p. 131) of these arrows as “transformations, processes, or ways of accessibility”.

16 Moreover, every set theoretic model $\mathfrak {S}$ gives rise to a (trivially categorial) $\phi$-counterpart functor model $\mathfrak {M}_{\mathfrak {S}}$ as follows. Include an object $\mathfrak {w} \in obj(\mathfrak {M}_{\mathfrak {S}})$ for each $w \in W$, and include an arrow $\mathcal {F}: \mathfrak {w} \rightarrow \mathfrak {w'} \iff Rww'$. Each object will be regarded as arrow theoretic, trivially, by setting $obj(\mathfrak {w}) = \mathcal {P} D_{w} \cong arr(\mathfrak {w})$ with each object its own identity and morphisms only identities. Then, since each $\mathfrak {w}$ is a discrete category, *any* function $\mathcal {F}: \mathfrak {w} \rightarrow \mathfrak {w}'$ is a functor, so in particular $\mathcal {F}(x) = \{ y | \langle x,y\rangle \in C_{w,w'} \}$ is. When $\mathcal {F}(x) = \{y\}$ is a singleton object from $\mathfrak {w}'$, say $\mathcal {F}(x) = y$, and give the counterpart functor its widest reading: $\hat{\mathcal {F}} = \mathcal {F}$ for all $\mathcal {F} \in arr(\mathfrak {M}_{\mathfrak {S}})$. The interpretation $I_{\mathfrak {w}}$ and actual world are unchanged. These constructions are not inverse to one another.

17 Meaning: requiring reference only to objects and morphisms.

18 Some are not arguments against realism so much as against seemingly plausible principles determining the size of the plurality (e.g. Stephanou, 2000)

19 See Lawvere and Schanuel (2009, pp. 40–41.)

20 See Parsons (1974).

21 See Pruss (2001).

22 “We can therefore perform all the usual operations of set theory on the elements of a universe without the end result ceasing to be an element of the universe. [On peut donc faire toutes les opérations usuelles de la théorie des ensembles à partir des éléments d’un univers sans, pour cela, que le résultat final cesse d’être un élément de l’univers.]”—Artin et al. (1973), author trans., see also Murfet (2006).

23 A category is *locally* $\mathfrak {U}$-small if all the collections of morphisms between objects of the category are $\mathfrak {U}$-small. In so far as we are inclined to be realists only about morphisms, it is then really *local smallness* that is of interest.

24 Indeed, it is perhaps closer to the spirit of Forrest and Armstrong’s arguement that we just reject the idea that there is a set of *all* possible worlds, on Lewis’s account, rather than the rejection of some pluralities of some worlds.
